# Simulation of Nondilute Dendrimer Systems with the Bond Fluctuation Model

**DOI:** 10.3390/polym14245363

**Published:** 2022-12-08

**Authors:** Juan J. Freire

**Affiliations:** Departamento de Ciencias y Técnicas Fisicoquímicas, Facultad de Ciencias, Avenida de Esparta s/n, 28232 Las Rozas de Madrid, Spain; jfreire@invi.uned.es

**Keywords:** dendrimers, Monte Carlo simulations, bond fluctuation model, scattering, radial distribution function, structure function

## Abstract

Using the bond fluctuation model, we performed Monte Carlo simulations for solutions of generation 4 and 5 dendrimers with only an intermediate unit between the branching points at different concentrations, ranging from moderately dilute solutions to concentrated systems close to the melt behavior. This model may represent different real types of dendrimer families. We obtained the mean sizes, asphericities, displacement of units, scattering functions, radial distribution functions and structure factors. We compared the results obtained for the last two properties with much faster Monte Carlo simulations of point-like dendrimers using global potentials obtained through the study of binary interactions. The latter procedure provided good reproductions of these properties but failed in the reproduction of the scattering functions in the range of higher concentrations. In this range, the scattering function cannot be described as the product of the structure function and the form factor, because the intensity decreases when the density of the dendrimer units becomes more homogenous.

## 1. Introduction

Molecular dynamics with atomistic models has progressively become the main tool to investigate molecular systems and, specifically, complex systems composed of nanostructures or polymers. However, the bead model and the lattice model are still useful when it comes to capturing the general features associated with them in a simpler way, due to the possibility of studying the processes that may imply greater configurational changes as the building of some degree of global ordering in particular systems [1].

Dendrimers are nanostructures of polymer molecules that have a structure that shows a regular hyperbranching disposition of the units around a core. These molecules can usually be functionalized, and they offer a variety of applications based on their particular conformational properties, such as catalysis or drug transport [2]. Dilute solutions of these molecules have been extensively studied using a variety of numerical techniques. We can mention the lattice [3] and coarse-grained models used for Monte Carlo [4] and Brownian dynamics [5] simulations, or molecular dynamics. The latter simulations are usually performed with an atomistic model, admitting different degrees of protonization and including explicit solvents [6,7]. Using a coarse-grained model, molecular dynamics has been extended to nondilute dendrimer solutions of poly(propylene imide) dendrimers with a 1,4 diaminobutane core, or PPI-DAB molecules, reaching volume concentrations that may represent the melt behavior [8].

Traditionally, lattice models have been employed to perform Monte Carlo simulations of many-chain linear chains. These models are particularly efficient because the coordinates of the units are placed in a discrete number of lattice positions, which permits to explore more global configurational changes. The bond fluctuation model [9,10] (BFM) considers bonds with different orientations and lengths in a lattice that allows for more flexibility in the definition of the polymer structure while maintaining the simplicity of computation inherent to lattice models. We applied this model to study linear chains and star polymers [11]. The model has also been applied to dendrimers [12,13,14]. In particular, an investigation of the overlapping between units and radial distribution functions in nondilute dendrimer systems has been carried out using the BFM [14] for molecules with different spacers, or number of units between branching points, ranging from 4 to 32. This number of spacers allows for a high degree of internal flexibility so that the molecules can be easily generated within the lattice structure. However, models with particularly flexible segments between the branching points cannot represent the actual structure of the most commonly used dendrimer molecules. 

In this work, we report the results obtained for the BFM nondilute dendrimer structures of generations 4 and 5, *G*4 and *G*5, with a single spacer between the branching points. Potentially, these particular structures can be parameterized in order to characterize some of the most common dendrimer molecules, such as polyamidoamine dendrimers with an ethylendiamine core, or PAMAM-EDA, and PPI-DAB; though, in this work, we employed a generic model. We obtained the sizes, shapes and global displacements of the individual molecules in systems of different concentrations, ranging from moderately dilute solutions to concentrated systems. We also evaluated the radial distribution functions, form factors and structure factors. Moreover, we simulated the scattering intensities for the same systems. We performed a qualitative comparison of the scattering-related results of our generic model with those obtained previously for a coarse-grained model of real PPI-DAB dendrimers using the molecular dynamics method [8]. As in the analysis of the latter results, we also considered in our discussion some neutron scattering experimental data corresponding to the same dendrimers [15,16]. We mainly focused on the variation of the scattering function with the concentration. In particular, we discuss the validity of the factorization of this function in terms of the form factor and the structure factors when the concentration increases. We also investigated the accuracy of the radial distribution functions and the structure factors obtained with much faster Monte Carlo calculations carried out with a global model of point-like dendrimers, using a potential derived from the study of binary interactions between the BFM molecules.

## 2. Numerical Methods

According to the BFM specifications [9], each bead occupies a lattice site blocking the neighboring 26 sites of a simple cubic lattice. This restriction fulfills the self-avoiding walk (SAW) condition so that the minimal distance between units corresponds to 2 (the distance unit is defined as the distance between neighboring lattice sites). The bond lengths are chosen from all possible connections between the sites in a range between 2 and $\sqrt{10}$, but bonds of the type (±2 ± 2 0) and the equivalent are avoided, because they may cross each other during the simulation. We assigned different energies to the different types of bonds [17], but we did not associate energies to close the interacting non-neighboring units. The model only considers long-range repulsive interactions represented by the SAW condition and, therefore, it describes dendrimers immersed in a good solvent.

**Figure 1:**
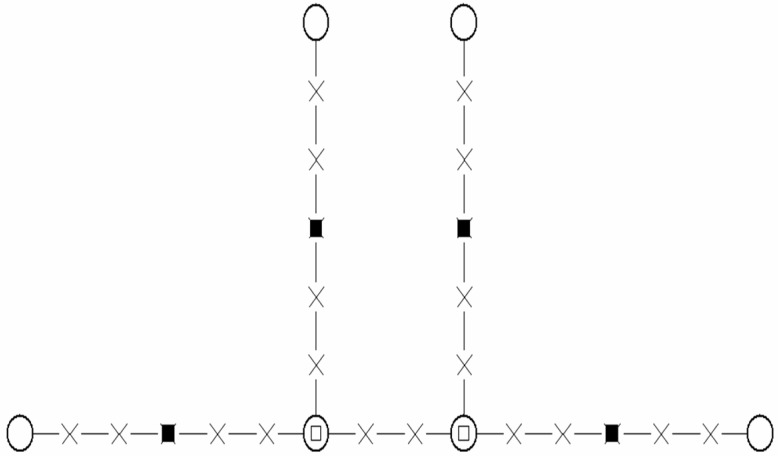
Initial disposition of the core, *G*0, units. Crosses: lattice points; black squares: intermediate units; circles: branching points; circles with squares: central units.

We built initial conformations of the dendrimer molecules consistent with the self-avoiding condition. For the initial configuration, we defined two neighboring centrals joined by a (±3 0 0) bond and connected each of them with two new bifunctional branching units to form the core (or *G*0 dendrimer) (Figure 1). We added new generations up to *G*4 or *G*5 in a similar way. Each one of the segments connecting successive branching points contained two identical BFM bonds. Moreover, the unit positions in the core were simply selected to avoid overlapping, while the branching point positions in other generations were determined by randomly choosing the two identical BFM bond vectors that define their connection with the previous branching unit. We included an intermediate unit between every pair of successive branching points in the middle site connecting the two successive vectors. Therefore, the total number of units was *N* = 250 and *N* = 506 for the *G*4 and *G*5 dendrimers. A conformation was rejected if the nonoverlapping condition was not fulfilled after we defined a new branching unit position, and new attempts were performed until we found a conformation without overlaps. We verified that this procedure could only be applied to generate *G*5 or smaller generations of our particular BFM dendrimer structures.

When the mean value of the distances between the branching units, between 4 and 2$\sqrt{10}$, was compared with the minimum distance between the nonoverlapping units, 2, the ratio roughly corresponded to that found in some of the most common dendrimer molecules. Thus, for the molecular dynamics calculations for PAMAM-EDA, the molecules showed a most probable distance between the branching units of 7.5 Å, and they closely reproduced their experimental sizes with a coarse-grained model if a value of 2.9 Å was set for the overlapping distance between the beads [18]. Using the ratio of the overlapping distances for the PAMAM-EDA coarse grained and the BFM to scale the BFM distances to real values, we obtained a mean distance between the branching points for the PAMAM-EDA molecules of (2.9 Å/2) × 5.2 = 7.5 Å. Therefore, the BFM seems to be an adequate representation for one of the most common dendrimer families. Moreover, a better refinement is possible, since the probabilities of the BFM bond distances can be fitted for a better representation of the real distances of particular dendrimers. Carefully chosen modifications of the bond energies may lead to changes in the averaged distance between the neighboring dendrimer units that presumably yield a better consistency with the experimental sizes of real dendrimer molecules. However, though one of the motivations of this work was to show that this can actually be achieved, and our size results showed a rough agreement with the PAMAM-EDA data, we have not specifically addressed any particular fit for real dendrimers. Instead, we chose to use the original version of the bond energies to check if this description can be adopted for initial, non-refined structures in order to perform the characterization of different real dendrimers. We also remark that adding more intermediate units [12,13,14] improves the flexibility of the molecules, allowing for a faster equilibration, but it gives less realistic descriptions of the most common dendrimers families.

We built a simulation box introducing the molecules in regular arrangements to fulfill the required densities and considering the maximum possible distance between them for each given density to facilitate equilibration. However, we verified that the most congested systems, corresponding to the highest concentrations of *G*5 dendrimers, cannot be properly equilibrated using this initial disposition of molecules. When all of the nearly located molecules are close and cannot find any preferred direction to advance, they cannot interpenetrate easily in a given direction and tend to order, leaving regions of lower density outside the sphere-like shaped molecules. In order to solve this problem, we added a space restriction to the conformations along two axes. Particularly, we used elongated conformations, or “rods”, that only extended 8 lattice sites along these axes. They were similar to the initial conformations that we previously used for star molecules [11]. These conformations with more initial open space in a given direction are able to reach their equilibrium near to spherical shape, while they also can interpenetrate, leading to a close to homogeneous density of dendrimer units in the case of the most concentrated systems.

We introduced *n_t_* molecules, each of *N* units, in a cubic lattice of length *L* to create the different systems at the desired concentrations. Initially, the box lengths were set to be in the range *L* = 96–108. These values allowed us to avoid interactions between the replicas of any dendrimer unit generated by the application of periodic boundary conditions. The properties of the individual molecules were obtained from these simulations. However, these box sizes were found out to be only approximately 2.5 times the dendrimer mean diameters. Consequently, we made final runs with boxes that duplicated these lengths in order to obtain the global properties involving the molecule positions. *n_t_* was fixed to comply with a fixed fraction of the occupied sites, or volume fraction, given by Φ = *n_t_M*/*L*^3^, where *M* is the total number of sites blocked by the molecule units. Since 27 sites were blocked around a single lattice unit in the BFM, a unit effectively occupied 8 sites and, therefore, *M* = 8 *N*. We obtained the results for three different volume fractions. 

Starting with the configurations obtained with the described methods, we ran the simulations over a given number of Monte Carlo bead jumps. A bead jump consisted of the displacement of a single unit to one of the blocked neighboring sites. The jump was accepted if it complied with the bond distance specifications and was able to avoid overlaps with respect to the nonbonded units, SAW condition. We used the Metropolis criterion [19], considering again the previous configuration in the case a jump was rejected. We defined a simulation step as *n_t_N* jump attempts, giving each unit a single statistical possibility to move. We introduced a certain number of initial equilibration steps in all cases (at least 10^6^ steps), and we collected the properties every 4000 steps once the size and asphericity fluctuated around a constant mean. We ensured that the size and asphericity values did not vary in more than 1% after two successive runs. In some cases, we needed to extend our simulations to 10^8^ time steps. The global properties were evaluated as averages over the configurations contained in a final equilibrated run.

**Figure 2:**
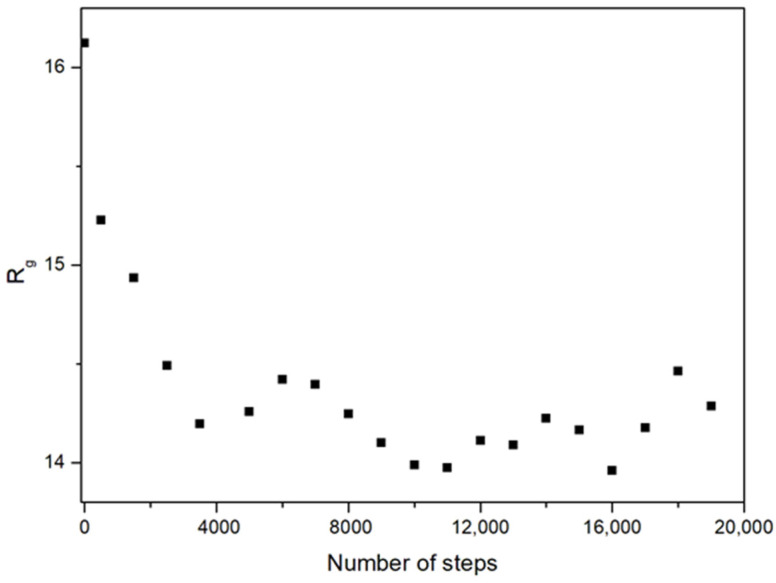
Averaged values of the dendrimer size obtained at different steps of the trajectory for the G5 dendrimer, Φ = 0.2 system. The averages were taken every 1000 steps. The mean size in the first configuration is also included as a reference.

We characterized the dendrimer size as the root mean squared radius of gyration, *R_g_*, and also as the root mean squared distance between the center of the dendrimers and the units belonging to the outer generation, *R_ce_*. In Figure 2, we show the size equilibration curve for one of the systems. The deviation from a spherical shape is shown in terms of the asphericity [20,21]:

(1) $$A=\frac{<\sum_{i>j}^{3}{\left(\lambda_{i}-\lambda_{j}\right)}^{2}>}{<2{\left[\sum_{i=1}^{3}\lambda_{i}\right]}^{2}>}$$

where λ*_i_* is the *i*th eigenvalue of the radius of the gyration tensor.

We also obtained the squared displacement of the center of the masses along the trajectories $\langle R_{cm}^{2}\rangle$. The variation of $\langle R_{cm}^{2}\rangle$ with the number of steps in the linear regime allowed us to provide estimations of the apparent dendrimer diffusion coefficients as dimensionless values that could be proportional to the experimental values of the actual diffusion coefficients for different real systems if the model molecules were able to behave as in a true diffusional regime. 

Furthermore, we obtained a numerical estimation of the scattering intensity as:

(2) $$I\left(q\right)=\left(8/L^{3}\right){\left[\sum_{i}^{L^{3}}f_{i}cos\left(q.R_{i}\right)\right]}^{2}+{\left[\sum_{i}^{L^{3}}f_{i}\sin\left(q.R_{i}\right)\right]}^{2}$$

where **q** is the scattering vector, and **R***_i_* is the vector defining the position of lattice site *i* within the box; *f*_i_ describes the occupation of the site, adopting the value *f_i_* = 1 − Φ/8 if it is occupied by a dendrimer unit and *f_i_* = −Φ/8 if it is empty. We also evaluated the form factor for the individual molecules, *P*(*q*), using Equation (2) for systems with the same box size but only occupied by a single dendrimer, taking into account the volume fraction and number of units of the molecule as:

*I*(*q*) = *ΦNP*(*q*) (3)

The positions of the dendrimer center of masses can be used to obtain the radial distribution function, *g*(*R*), for the different systems. To this end, we obtained the averaged histograms of the number of molecules with centers of masses that were at different distances from that of each given dendrimer. The structure factor, *S*(*q*), is simply related with the Fourier transform of *g*(*R*). It can also be obtained from the relative position between the centers of the masses:

(4) $$S(q)=\left(1/n_{t}\right)\langle \sum_{i}^{n_{t}}\sum_{j}^{n_{t}}e^{-iq.R_{ij}^{cm}}\rangle$$

Since *g*(*R*) and *S*(*q*) do not depend on the coordinates of particular dendrimer units, we explored the possibility that they can be calculated using point-like dendrimers that interact through a global intermolecular potential [22]. We previously used this procedure to obtain the structure function of dilute systems of PAMAM-EDA and PPI-DAB dendrimers to obtain the scattering intensity of dilute PAMAM-EDA molecules [23] using the factorization approximation that is described below. Following a method initially proposed to study n-alkane chains [24], the global intermolecular potential was obtained by calculating the conformational and orientational average of the Mayer function corresponding to the intermolecular interactions between the units of a pair of molecules, placed at given distances between their centers of masses, *R*:

(5) $$fM(R)={\langle \exp\left(-U_{\mathrm{inter}}\left(R,\theta ,\Phi \right)/k_{B}T\right)\rangle }_{\mathrm{conf},\theta ,\Phi }-1$$

In order to perform this average, we considered randomly chosen pairs of individual conformations obtained in the simulation of the single BFM dendrimer systems employed for the calculation of the form factor. We distributed 10^5^ tries of binary interactions among 200 intervals of values of *R* in a range where we expected all of the different cases of partial overlapping to be contained according to the known radius of the gyration values. Each one of the intervals included a minimum number of 26 values of *R*. We randomly chose the orientations that corresponded to every different value of *R*, determining the angles θ and Φ and, consequently, also defining the vector *R* that connected a pair of conformations. These conformations were randomly chosen among those generated in the trajectory. We collected 4500 data, determining how many pairs of chains showed any intermolecular overlap of units and, therefore, contributed to the Mayer function. From these results, we obtained an effective potential as:

(6) $$w_{eff}(R)/k_{B}T=-\ln\left[1+f_{M}\left(R\right)\right]$$

**Figure 3:**
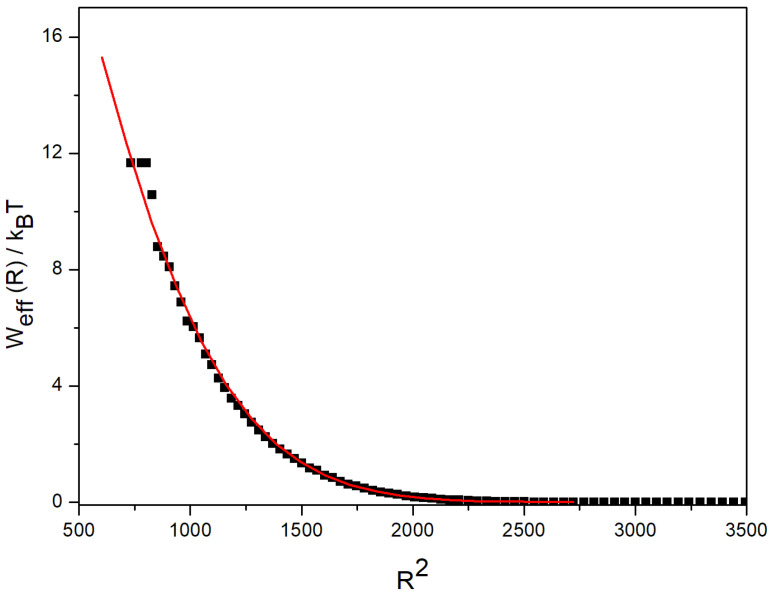
Effective potential for the *G*5 dendrimer. Black symbols: results obtained from Equations (5) and (6); red line: fit as specified in the text.

This potential had a decay shape, see Figure 3, for the *G*5 results.

The logarithmic representations of the decaying potential function vs. *R*^2^ were well fitted to second degree polynomials, which allowed us to obtain the analytical interpolation functions. It should be mentioned that a Gaussian shape has been successfully proposed to represent the effective potential for low generations of dendrimers [25,26]. However, we verified that the Gaussian form cannot accurately describe the somehow harder effective binary intermolecular interactions found when the generation number increases. Similarly, more complex forms that imply harder intermolecular interactions have been found [27] using a model that also includes intermolecular attractions.

Our resulting fitted functions were employed in subsequent Monte Carlo simulations for point-like dendrimers to obtain estimates of *g*(*R*) and *S*(*q*) using bigger simulation boxes. The method was checked by comparison of its results with those obtained with the BFM with dendrimers composed of interacting units. The Monte Carlo simulations with point-like dendrimers also considered the Metropolis criterion and periodic boundary conditions, using the same number of molecules and box lengths employed for the BFM systems. We performed 10^7^–10^8^ randomly oriented single jump displacements of coordinates between 0 and 0.5, collecting the properties every 1000 steps. This procedure avoids the spatial restrictions inherent to the lattice models. When compared with the BFM model results of the properties related to the disposition of the molecules’ center of masses, as the radial distribution function, we can check the influence of these restrictions by the presence of dendrimer units. In the cases where no significant differences were found, the agreement supports the validity of the simulation methods. We remark that the use of point-like molecules imply much faster calculations, since we only have to deal with *n_t_* interacting particles, while the BFM simulations considered interactions between *n_t_N* interacting units.

## 3. Results and Discussion

In Table 1, we present the results obtained with our BFM simulations for the dendrimer size in terms of *R_g_* and *R_ce_* and also for the asphericity. In the case of flexible chains, the overlapping concentration is usually estimated from the mass of the molecule and its radius of gyration in dilute conditions, Φ* = *M*/(4 π $R_{g}^{3}$/3). However, as described above, dendrimer molecules cannot easily interpenetrate due to the congestion of the units forming the outer shell and, in order to avoid overlapping, they simultaneously tend to adopt ordered dispositions. If we represent the dendrimers as hard spheres of radius *R_ce_*, the maximum possible density without overlapping is given by Φ*_over_* = 0.74 *M*/(4 π $R_{ce}^{3}$/3). Factor 0.74 corresponds to the occupation factor in the densest disposition of tangent spheres. The values for this density are included in Table 1. Comparing the considered densities with the corresponding Φ*_over_* values, we observed that our lowest volume fractions, Φ ≅ 0.05, was well below Φ*_over_* and, therefore, most molecules were isolated, and they did not need to be ordered. The intermediate value, Φ ≅ 0.1, represents more concentrated systems with densities approximately 65–70% of the densest packing; molecules may partially order or overlap. The highest value fraction, Φ ≅ 0.2, was greater than Φ*_over_* for both generation numbers and, therefore, there must be some degree of overlapping between molecules, even if the system is ordered.

It should be mentioned that the melt state in the BFM cannot correspond to Φ = 1, a value that does not allow for any motion of the units. The alternative value, Φ = 0.5, was considered as a more accurate representation of a BFM melt [28]. In our recent calculations for symmetric star polymers, an even smaller value, Φ ≅ 0.36, was consistent with the melt behavior [11]. Therefore, the highest volume fractions in the present study corresponded to considerably concentrated systems not too far from the melting state.

We can observe that the size decreased with the increasing volume fraction for each given generation, as in the case of common polymers in good solvents, due to the screening of the excluded volume. The ratio between the center-to-end distance and the radius of gyration did not depend on the concentration, and it was 1.15 and 1.11 for the *G*4 and *G*5 dendrimers, respectively. These values can be compared with the theoretical limits for a compact sphere, (5/3)^1/2^ = 1.29, and for an unperturbed polymer coil, 3^1/2^ = 1.73. The density within the dendrimers increased towards the outer shells for which the distance to the center was the greatest. This explains why these dendrimer ratios were smaller than those of the polymer coils, and they were even smaller than those of the compact spheres.

Taking the ratio (2.9 Å/2) described above to scale the BFM lattice distances to real units, we obtained values of *R_g_* = 18.9 Å and 23.2 Å for *G*4 and *G*5 in the most diluted systems. These results are not far from the neutron scattering experimental results obtained for non-protonated PAMAM-EDA polymers in neutral dilute solutions, *R_g_* = 21.4 Å and 25.5 Å [29,30,31]. We should remark again that the distance-dependent bond energies could be varied to refine the simulation results for particular types of dendrimers. An adequate refinement may also lead to good representations of other specific real dendrimers such as PPI-DAB. However, in this work, we focused on the results obtained with the set of energy bonds originally proposed [17].

The small values for the asphericity are close to the spherical limit, *A* = 0. The asphericity did not significantly change with the volume fraction for the most diluted *G*4 dendrimers, though a small increase was observed for the highest concentration. However, a small but clearly progressive increase was noted for the *G*5 molecules. A small increase in the asphericity has also been observed for BFM systems of highly branched star polymers and higher concentrations but the opposite tendency seems to be observed for the case of BFM linear chains and lightly armed stars [11], in accordance with the results obtained with a simple cubic lattice model [32]. Therefore, the observed slight deformation with respect to the spherical shape seems to be associated to the increasingly more difficult overlap of the more compact molecules. This contrasts with the behavior observed in the case of polymers with a more uniform density profile for which interpenetration can be easily achieved.

**Figure 4:**
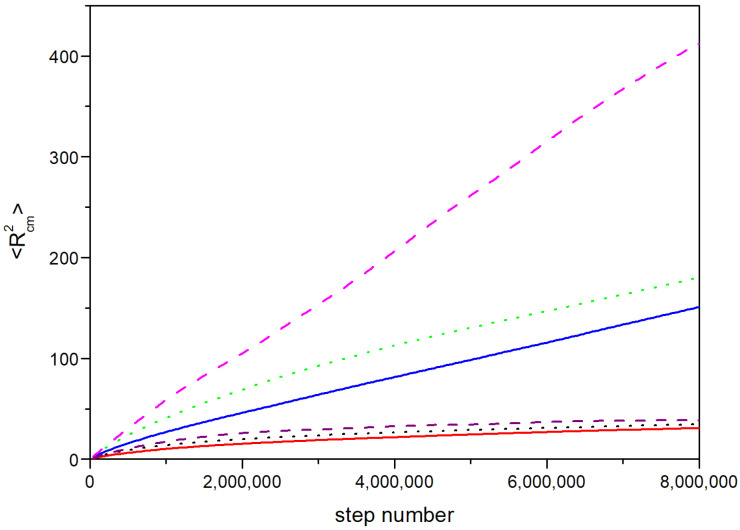
Averaged displacement of the dendrimer center of masses. Lower curves, *G*5; red solid: Φ = 0.2; black dot: Φ = 0.09; purple dash: Φ = 0.05. Upper curves, *G*4; blue solid: Φ = 0.17; green dot: Φ = 0.08; magenta dash: Φ = 0.04.

In Figure 4, we show the mean quadratic displacement of the molecular center of masses for the different systems. Assuming a linear behavior for the global squared displacement of the molecule versus the number of steps along the trajectory, an apparent diffusion coefficient was evaluated as one-sixth of the numerical values of the slopes obtained by fitting the average quadratic displacement curves in the range of the step number values where the linear regime was observed. These values are included in Table 1. It was observed that the displacements were small for the *G*5 systems, though they reached the linear dependence in all cases. The apparent diffusion coefficients were similar for the three concentrations, and their values seemed to be mainly associated with the dendrimer sizes, since there were not important differences due to the concentration for a given generation. However, the initial nonlinear displacement was slower for the denser systems. The *G*4 systems showed greater displacements than the *G*5 molecules. The denser Φ = 0.08 and Φ = 0.17 systems showed similar slopes, but a significantly higher value was found for the Φ = 0.04 system. It should be remarked that the decrease in the global displacement for the *G*5 molecules, containing a greater number of units, with respect to the *G*4 dendrimers, was considerably greater than the *N*^2^ dependence theoretically predicted and experimentally verified for entangled flexible polymers. This reveals the greater difficulty of mobility of the bigger dendrimers due to the fact of their particularly rigid structure. Although internal equilibration of the molecules can be accomplish relatively easily, the global properties involving molecular displacements may require an extra equilibration effort and efficient molecular models.

**Figure 5:**
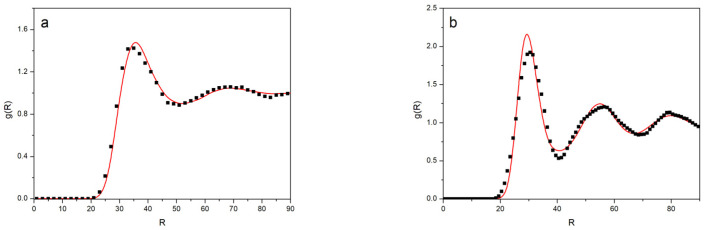
Radial distribution functions: (**a**) *G*4, Φ = 0.04; (**b**) *G*5, Φ = 0.2. Black symbols: from the BFM results; red lines: from the point-like dendrimers interacting through a global potential.

The radial distribution functions were obtained from the BFM simulations and from the much faster Monte Carlo simulations of the point-like particles for the different dendrimers and concentrations. We compared the results obtained with these two different types of simulations in two different systems. In one of them, the molecules could move freely, allowing for a faster equilibration (*G* = 4, Φ = 0.079), while the other showed more restricted motions (*G* = 5, Φ = 0.20) (see Figure 5). It can be observed that the agreement was excellent for the more dilute system. Moreover, the fast method provided a good estimate, even in the case of the more concentrated and restricted system. Therefore, the Monte Carlo simulations performed with a global potential for a point-like model of dendrimers seemed to provide a reasonable estimation of the radial distribution functions for all of the different dendrimer systems. Our curves for the concentrated *G*5 system had several maximum values, revealing a somehow ordered structure. They were in qualitative agreement with the radial distribution function obtained for a melt of PPI-DAB in a molecular dynamics simulation that used a coarse-grained model [8].

We also obtained the structure factors from the simulations using the BFM and the point-like dendrimer models. Since *S*(*q*) is directly related with the Fourier transform of *g*(*R*), we can expect a similar agreement with the results obtained from both methods. However, the BFM results directly calculated for Equation (4) had to be smoothed to agree with those evaluated from *g*(*R*) due to the notable oscillations around the peak values. It is possible that the discrete number of positions and the *q* values inherent to the BFM model were responsible for this feature.

The factorization approximation, used for dilute solutions, was based on a generalization of Equation (3) for systems with more than one molecule that take into account the structure functions to describe the effect of intermolecular interferences:

(7) I(q)=NΦP(q)S(q)

**Figure 6:**
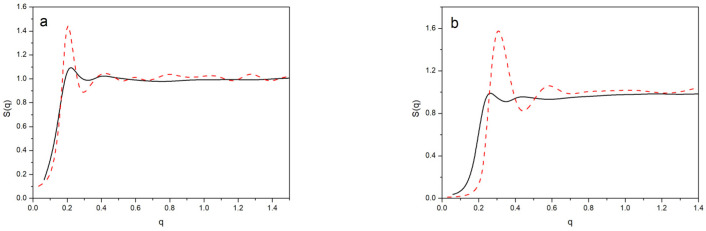

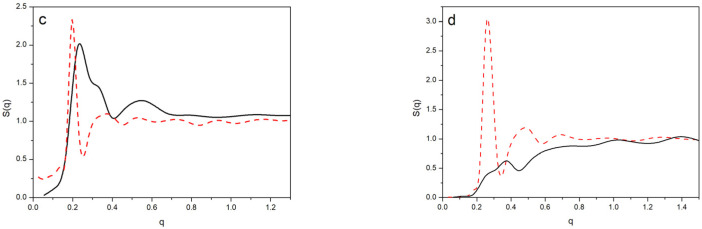
Structure factor for different systems: (**a**) *G*4, Φ = 0.04; (**b**) *G*4, Φ = 0.17; (**c**) *G*5, Φ = 0.09; (**d**) *G*5, Φ = 0.2. Red, dashed lines: from point-like dendrimers interacting through a global potential; black solid lines: from the total intensity and form factor, Equation (7).

In Figure 6, we plotted the results for the structure factor obtained directly from the simulations, Equation (2), and also the values of *S*(*q*) calculated from *I*(*q*) and *P*(*q*) in accordance with Equation (7). We observed that the latter equation provided a rough description of *S*(*q*) for the simplest systems, the lower generation and more dilute systems. However, the apparent structure factors obtained this way showed a significantly smoother variation with nonprominent peaks and a progressive increase to the asymptotic value for the most concentrated systems. It should be considered that, in the highly concentrated systems, the molecules were forced to overlap and, therefore, there was a near to homogeneous density profile of the dendrimer units at the distance range explored by the experimental values of *q*. This implies low and flat scattering intensities. However, the centers of masses were distributed in a somehow ordered form. This is the feature mainly reflected by the structure factors and, according to our results, it can be correctly described by the global point-like dendrimer model, even in the cases where molecules overlap.

The same features were previously reported in an analysis of the results of coarse-grained molecular dynamics simulations compared with experiments [8] for non-dilute PPI-DAB dendrimers. Our results seem to agree with those derived with a particular set of neutron scattering data [15] for the same dendrimer: clearly tending to unity for the highest values of *q*. However, the molecular results from the dynamics simulation and other experiments [8,16] showed a smaller increase in the structure function, with values that remained too low, even in the high *q* region.

**Figure 7:**
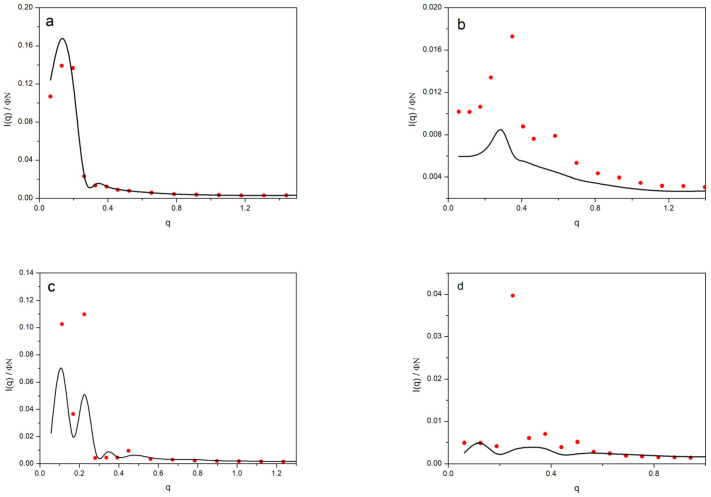
Normalized scattering intensity for different systems: (**a**) *G*4, Φ = 0.04; (**b**) *G*4, Φ = 0.17; (**c**) *G*5, Φ = 0.09; (**d**) *G*5, Φ = 0.2. Black lines: intensities from the BFM results; red circles: intensities from the structure factors obtained with point-like dendrimers interacting through a global potential and form factors, Equation (7).

The form of the scattering functions is illustrated in Figure 7, where the data obtained directly from the BFM model and the results obtained for *S*(*q*) via Equation (7) were included for an alternative analysis. It should be mentioned that the neutron scattering data for the PAMAM-EDA dendrimers in the dilute neutral solutions showed a range of roughly constant intensities for small values of *q* [30]. This feature was due to the cancelation between the decrease in *P*(*q*) and the similar increase in *S*(*q*). These scattering intensities showed a decrease for the greater *q* values. Actually, these data were reasonably well described by Equation (7) using the *S*(*q*) values obtained from our faster simulation method and *P*(*q*) [23]. However, the scattering curves for the higher concentrations, obtained in this work, showed peaks. The curves obtained from Equation (7) showed pronounced peaks, while considerably flatter curves were obtained directly from the BFM simulations. This was due to the increasing homogeneity in the density of units in concentrated systems as dendrimers are forced to overlap. Therefore, the description of the scattering intensities for the nondilute systems could not be performed with the point-like global potential, since this model is unable to adequately describe the correlation between the dendrimer units. In the particular case of the *G*5, Φ = 0.2, system, the intensity curves directly calculated from the BFM system showed a very smooth *I*(*q*) curve, with small values and fluctuations, behavior that was similar to that expected for a melt. However, when we approximate *I*(*q*) according to Equation (7), the results showed a high and sharp peak due to the regular disposition of the centers of masses described by the structure factor. Consequently, Equation (7) constituted a very poor estimation to predict the scattering intensities in this particular case and the point-like model was not useful to describe this property.

Finally, we should point out that the present BFM without an attractive interaction between units can only represent neutral dendrimers. In the particular cases of the PAMAM-EDA and PPI-DAB families, the molecules are protonated in solutions with a low pH. Although only moderate changes were observed for the size and shapes of these molecules, the scattering intensities in a dilute solution of protonated dendrimers were dramatically different, exhibiting peaks due to the long-range interactions between the charged molecules instead of an initial range of nearly constant values [23,29,30,31,33]. These differences are expected to decrease for the more concentrated systems, especially for the volume fractions approaching the melt where the molecules were closer, and the solvent role was severely diminished.

## 4. Summary and Conclusions

Nondilute systems of *G*4 and *G*5 dendrimers were simulated using the BFM. These dendrimers contained only a single intermediate point between the branching points to approach the structure of some of the most common real dendrimer systems. Actually, when the results for the dendrimer sizes were mapped to those of the PAMAM-EDA dendrimers, we found that the results were not far from the experimental data. Other properties, such as the global quadratic displacements of the molecules, asphericities and radial distribution functions, were also calculated. The asphericities were close to the spherical shape limit and showed the variation with the concentration expected from previous studies. In the case of the largest dendrimer, we observed a restricted mobility. The radial distributions were close to those obtained with alternative and much faster Monte Carlo simulations for point-like dendrimer particles interacting with a potential obtained by evaluating the binary interactions between pairs of BFM molecules, even for the highest generation and the more concentrated systems. We also reported structure factors and scattering intensities for these systems. In accordance with previous neutron scattering experimental results and simulations with a coarse-grained model in the open space, we observed that the scattering intensities could not be obtained from the structure factors of the systems and the form factors of individual molecules according to the factorization approximation for the more concentrated systems. These intensities were small when the systems reached concentrations for which there was a significant interpenetration between dendrimers implying a homogeneous distribution of units. However, the high peaks observed in the structure factors indicate that a substantial degree of order in the position of the dendrimer molecules developed simultaneously and competed with their mutual overlapping. The point-like model is useful to describe this feature, though the scattering intensities can only be correctly obtained with the BFM model in the case of nondilute systems.

## Figures and Tables

**Table 1:** Values of the dendrimer size, asphericity, overlapping assured volume fraction and apparent diffusion coefficients, as defined in the text, for the different dendrimer systems. Distance units correspond to the neighboring lattice site separation. The units of the apparent diffusion coefficients correspond to squared distances divided by the number of steps.

	Φ	*R_g_*	*R_ce_*	*A*	Φ_over_	*D* × 10^6^
*G4*						
	0.041	12.9	14.8	0.048	0.109	8.8
	0.079	12.6	14.5	0.048	0.115	2.8
	0.171	11.7	13.5	0.054	0.143	2.8
*G5*						
	0.048	16.0	17.7	0.028	0.128	0.35
	0.090	15.6	17.4	0.037	0.138	0.35
	0.202	14.1	15.7	0.048	0.184	0.43

## Data Availability

Not applicable.
